# Advanced nurse practitioners in municipal healthcare as a way to meet the growing healthcare needs of the frail elderly: a qualitative interview study with managers, doctors and specialist nurses

**DOI:** 10.1186/s12912-017-0258-7

**Published:** 2017-11-16

**Authors:** Birgitta Ljungbeck, Katarina Sjögren Forss

**Affiliations:** 1 Municipal Healthcare in Hässleholm, Management of Care and Welfare, Hässleholm, Sweden; 2 Department of Care Science, Faculty of Health and Society, Malmö University, -205 06 Malmö, SE Sweden

**Keywords:** Advanced nurse practitioner, Content analysis, Doctors, Manager, Municipal healthcare, Qualitative research, Primary care, Specialist nurse

## Abstract

**Background:**

The number of frail elderly people with complex nursing and medical care needs is increasing, and consequently, the healthcare burden. The implementation of Advanced Nurse Practitioners globally has been shown to make healthcare more effective and increase patient safety, continuity of care and access to care. In Sweden, research about Advanced Nurse Practitioners is limited. Thus, this study aimed to investigate the opinions of managers, doctors and nurses in primary care and municipal healthcare about the role of ANPs in municipal healthcare as a way to meet the increasing healthcare needs of the frail elderly.

**Methods:**

Managers, doctors and specialist nurses in primary care and municipal healthcare adopted a qualitative, descriptive design through 12 semi-structured interviews. The data were analysed using content analysis.

**Results:**

The participants expressed both opportunities as well as challenges with Advanced Nurse Practitioners in municipal healthcare. This role considered to satisfy frail elderly people’s healthcare needs and making the healthcare more effective as the doctors would have more time for other patients. The challenges mainly consist of doubts from the managers whether the nurses would be motivated to pursue further education to become an Advanced Nurse Practitioner if the role becomes a reality. The doctors were unsure if the nurses would consider taking the responsibility the role would imply.

**Conclusions:**

Advanced Nurse Practitioner is considered to be a valuable resource not only for the frail elderly but also for the nurses in the municipal healthcare and for the doctors in primary care as they probably would make healthcare more effective. They might be a way to meet the increasing healthcare needs of frail elderly, however there are also challenges to overcome before they can become a reality in a Swedish healthcare context. Consequently, this role deserves further investigation.

**Electronic supplementary material:**

The online version of this article (10.1186/s12912-017-0258-7) contains supplementary material, which is available to authorized users.

## Background

The Swedish healthcare system currently faces major challenges. One of the most pressing is to improve the efficiency of healthcare for the growing group of the frail elderly [1, 2], meaning those who are at an increased risk for hospitalization and dependency, and who have a reduced life expectancy [3]. The shortage of doctors in primary care affects the continuity of care as well as the availability of healthcare for this vulnerable group. It also affects the nurses who work in municipal healthcare, as they find it difficult to make consultations with the doctors’ due this shortage [1, 2]. This is a risk to the patient safety and may also lead to unnecessary hospitalizations of the frail elderly [2].

One way to address these challenges may be to educate and implement Advanced Nurse Practitioners (ANPs) in the municipal healthcare service [4]. ANPs was introduced in the USA during the 1960s and is now a well-established and essential nursing profession in many countries such as Holland, Ireland and Great Britain [5]. International Council of Nurses (ICN) defines ANP as “A registered nurse that has the expert knowledge required, the ability to make complex decisions and clinical competence for an expanded work description, whose character is formed by the context and/or the country where he/she has the right to work” [6]. The development of the role of ANPs has resulted from difficulties recruiting enough doctors to meet increased healthcare demands [7]. Studies regarding evaluations of ANPs have shown increased patient safety as well as improved continuity and access to healthcare, which has made healthcare more efficient [8–10]. However, the unclear role ANPs have in the healthcare system [11–13] as well as doctors feeling their authority is being threatened has contributed to difficulties in implementing the role globally [14–16].

In Sweden, no established education exists to train to an ANP, therefore, the role is not clearly defined or legislated as a protected title [13, 17]. Nevertheless, the universities of Linköping and Skövde have trained a small number of ANPs, specialised in surgery care or primary care. In their role, they discharge patients from hospital, follow-up test result and write referrals for various health examinations, which has historically been the responsibility of a medical doctor [18–20]. In the Swedish context, studies have cited the limited authority for ANPs to prescribe medicine as a negative factor for the development of their role [14, 18, 19]. In Sweden, registered nurses may have the authority to prescribe certain types of medication if they have completed a supplementary pharmacology course which differs depending on whether one is a registered or a specialist nurse [21]. A Swedish registered nurse holds a Bachelor’s degree with 180 education credits. In Sweden, a national credit system is used by the universities to show the scope of a course or a study program. One week’s full-time study (40 h) corresponds to 1.5 higher education credit. One (1) Swedish credit is equal to one (1) ECTS (European Credit Transfer System) credit. After having a Bachelor’s degree, a specialist nurse must have completed 60–75 education credits on advanced level and have a 1-year Master’s degree [22]. Compared to a specialist nurse, an ANP generally has a 2-year Master’s degree with higher skills in advanced nursing and medical care [5, 23, 24] and usually with the extended authority to prescribe medication [25]. The extended authority to prescribe medication is a key factor in the ANP role; this makes it possible to assess and treat patients independently of doctors, which contributes to more effective healthcare [25]. The frail elderly, with their complex needs for advanced nursing and medical care, require high skills among nurses, and studies have shown ANPs as valuable when caring for this group [26, 27].

The numbers of frail elderly are set to increase, both in Sweden as well as in the rest of the world. According to the World Health Organization, the global population of elderly people aged 60 years or more was 600 million in 2000; it is expected to rise to around 2 billion by 2050 [28]. Indeed, a quarter to a half of people older than 85 years are estimated to be frail [29]. From a Swedish perspective, where the number of people who are aged 80 or older is expected to rise from about 500,000 in 2014 to 800,000 in 2030 [30], one must explore any opportunity to meet their growing healthcare needs. The healthcare system is not prepared for this demographic change; thus, a rethink of the system is necessary to meet this challenge [31]. The Swedish National Board of Health and Welfare have discussed whether the role of ANPs might be a way to make healthcare more effective to meet the growing needs of healthcare [1]. However, in Sweden, research about ANPs is limited. Thus, this study aimed to investigate the opinions of managers, doctors and nurses in primary care and municipal healthcare about the role of ANPs in municipal healthcare as a way to meet the increasing healthcare needs of the frail elderly.

## Methods

A qualitative, descriptive design was adopted to investigate the opinions of the participants. The data were collected through semi-structured interviews [32], and the subsequent content analysis was performed as described by Krippendorff [33].

### Settings

This study was conducted during winter 2016 in a municipality, defined as a suburban region, with about 50,000 inhabitants located in the southern part of Sweden. A relatively large proportion of the inhabitants live on the countryside and the main town of the municipality has about 18,000 inhabitants. In Sweden, primary care and municipal healthcare are usually separate organisations; nevertheless, both have a common responsibility for the frail elderly. The doctors in the primary care are responsible for their medical care, and the nurses working in municipal healthcare are responsible for their daily needs for nursing care. The frail elderly who cannot easily travel to primary care are entitled to receive healthcare at home. In municipal healthcare, each frail elderly person has their own contact nurse responsible for contact with a doctor in the primary care when their condition requires it [2]. In 2017, a new healthcare agreement began in the southern Sweden which means that more advanced healthcare is performed in frail elderly people’s homes instead of in hospital. This requires even more cooperation between doctors in primary care and nurses in municipal healthcare [34].

### Participants and recruitment process

The participants were chosen by a strategic selection [32]. To participate, the inclusion criteria were as follows: the managers must work in either primary care or municipal healthcare; the doctors must work in primary care but to some degree with registered or specialist nurses in municipal healthcare. Moreover, the specialist nurses must have worked in municipal healthcare as a specialist nurse for at least two years to have gained the experience and understanding that advanced nursing care requires. However, for the managers and the doctors, there was no such criteria.

The recruitment process started with contacting the managers from both primary care and municipal healthcare by email and then obtaining approval for the study. The researcher (BL) and managers from primary care then identified doctors who worked with nurses in municipal healthcare, and the researcher contacted them via email. The managers and specialist nurses who work in municipal healthcare were also found and invited by email. If they agreed to participate, the researcher (BL) contacted them to decide on a time for the interview which would take place at the participant’s workplace. Twelve informants accepted the request (Table 1). The participants comprised ten women and two men with a combined age range of 33–63 years. None of the nurses had an education as ANP and none of the participants had a collaboration or contact with ANPs. There is no ANP employed in the area where the participants of this study are working.

**Table 1:** Overview of the participants’ characteristics

Code	Profession	Workplace
A	Doctor	Primary care
B	Doctor	Primary care
C	Doctor	Primary care
D	Doctor	Primary care
E	Specialist nurse	Municipal healthcare
F	Specialist nurse	Municipal healthcare
G	Specialist nurse	Municipal healthcare
H	Specialist nurse	Municipal healthcare
I	Manager	Municipal healthcare
J	Manager	Municipal healthcare
K	Manager	Primary care
L	Manager	Primary care

### Data collection

The data was collected through semi-structured interviews, and an interview guide (Additional file 1) was created to meet the aim of the study. Before the interviews, two pilot interviews were performed [32]. These indicated that the questions were unclear, and the guide was revised. The interviews began with collecting demographic data and then followed an open question to investigate if the participants had any knowledge of ANPs. If not, the researcher (BL) gave a short summary about what the role means and how it acts globally. The questions that followed were *how? what?* and *why?* in relation to the first question and complemented with other probing questions [32]. The interviews lasted 30–50 min and were tape recorded and transcribed verbatim.

### Data analysis

The transcribed texts were analysed using content analysis, as described by Krippendorff [33], and focused on both manifest and latent levels. The transcribed texts were read several times to get a deeper understanding of the content, and any meaning units were identified and condensed without losing their essential content. The condensed meanings were coded and compared and any similarities or differences between the codes were recorded. Next, categories were created from the codes based on their similarities. Finally, six categories emerged which were placed into two main themes (Table 2). The analysis, done by the first author but continuously discussed with the co-author, was characterized by constant comparison of the categories with the original text to ensure that the interpretations were grounded in the data. The qualitative methods and reporting of results adhere to the COREQ (Consolidated criteria for reporting qualitative studies) [34] guidelines (Additional file 2).

**Table 2:** Examples from the analysis process reflecting opinions about ANPs in municipal healthcare as one way to meet the increasing healthcare needs of the frail elderly

Main themes	Categories	Codes
ANP as an opportunity	The role of ANP from the perspective to satisfy the frail elderly’s need of healthcare	Increased patient safety Better continuity More person-centred care Increased access to medical care at home
	The role of ANP from the perspective of making healthcare more effective	The nurses’ work would be easier Use the right skills to create efficient healthcare Prescription of drugs
	The role of ANP from the perspective of recruitment difficulties	Important to innovate for future recruitment challenges Career path for nurses to remain in patient care There are too few doctors
	The role of ANP from the perspective of increasing municipal nurses’ skills	Experience of low skills among some municipal nurses Need for nurses with more medical competencies in municipal healthcare New healthcare agreement
ANP as a challenge	The role of ANP from the perspective of collaboration between municipal healthcare and primary care	Create conditions for better collaboration between doctors and municipal nurses Need for nurses with support and advice from the doctor Confidence with the doctor Co-workers More time for the doctors to see other patients
	The role of ANP from the perspective of motivating nurses to study and the courage to take on the responsibilities that the role entails	Motivation among nurses to study as an ANP It must be worthwhile The employer must motivate higher education A tedious way for nurses with an older education to get authority for higher education Dares nurses to take a greater responsibility? Courage for nurses

## Results

Our findings illustrate opinions from managers, doctors and specialist nurses about the role of ANP in municipal healthcare as one way to meeting the increasing healthcare needs of the frail elderly. We categorised their opinions into two main themes: *opportunities* and *challenges*. The findings are exemplified with quotations from the participants.

### ANP as an opportunity

In this theme, opinions reflecting the role of ANP as an opportunity from four different perspectives are presented which may have a positive impact to meeting the increasing healthcare needs of the frail elderly.

### The role of ANP from the perspective to satisfy the frail elderly’s need of healthcare

The participants, especially the nurses, thought the role of ANPs would increase patient safety as the ANP would have the ability to put together several parts of a complex patient picture through their clinical competence, leadership and collaborative practice. The continuity of care was also considered to enhance as the ANP could follow the frail elderly through different types of services and take more responsibility for the patients regarding both nursing and medical care.

It would certainly increase the quality of care for our elderly to have this role. I think it will be safer if we [in the role of an ANP] have a more overall responsibility…and the continuity had become amazingly much better. (Specialist nurse/F).

The nurses stated that an increased patient safety through better continuity would mean a more personalised and person-centred care as the ANP would have in-depth knowledge of the elderly as individuals, not only as patients. The nurses also thought that the ANP would have more time to focus each patients situation as a whole that together with the ANPs advanced skills in knowing, doing and being the quality, safety, continuity and person centredness would improve.

I think that we have more time for the patient. We take time for the patient. We know the patient better, and we know when the patient needs a little extra care, so we take more time for this patient. (Specialist nurse/G).

### The role of ANP from the perspective of making healthcare more effective

The participants agreed that access to more healthcare in the frail elderly’s home would be improved with the role of ANP. The nurses believed this would save them time and make their work easier. Instead of spending time finding a doctor to consult, which was described as a tedious process, they stated that it would be easier if an ANP could do the entire assessment, including ordering blood sampling and prescribing medication. They also expressed that the frail elderly would be able to access healthcare more quickly.

It might be a bit quicker for the patient to get help. If there is anything, then you do not need to go through the health center and wait. But then, we [as ANPs] can be faster with the help, I think. (Specialist nurse/G).

The managers considered that ANPs could reduce the doctor’s workload with the frail elderly, so they could spend more time with other patients thus making the healthcare more effective. The doctors agreed that there were tasks that could be done more easily with the help of an ANP, as they often do not assess the patient (in the municipal healthcare) without acting after the nurse’s assessment, often via a telephone consultation.

I think it is about doing things easily. So, if you have conducted an assessment, so why should [the doctors] just click for the prescription? We [the doctors] go anyway on what you [the nurses] tell us. So, this is your [the nurse’s] assessment, right? We [the doctors] don’t go in and assess the patient. It is not better for the doctor to write out the antibiotic, I mean it may just as well have been by the nurses. (Doctor/D).

### The role of ANP from the perspective of recruitment difficulties

The participants agreed that the healthcare sector faces difficult recruitment challenges to meet the increasing healthcare needs of the frail elderly. The manager from municipal healthcare said that they had major difficulties in recruiting nurses with the right competencies and in retaining competent nurses. The manager and the nurses thought a key reason was that the nurses do not see any career opportunities in municipal healthcare, and consequently, do not consider this work attractive. However, they believed the role of ANP might be a way to have a clinical career and continue to work in patient care.

When I got familiarized with this [the role of ANP] I realized that it could be an opportunity to make a step in the career, and then many [nurses] might have stayed (Specialist nurse/E).

The managers from primary care have also had difficulties recruiting doctors. Accordingly, they said it was important to be innovative in meeting the increasing healthcare needs of the frail elderly, as the doctors were not able to work effectively with their current resources.

There is such an enormous shortage of doctors, and if we look at our field of General Practitioners, we must do something. I think it is important and am totally convinced that if we are to get anywhere, we must come up with new things … [aim to the role of ANP]. (Manager, primary care/K).

### The role of ANP from the perspective of increasing municipal nurses’ skills

Both managers and doctors stressed that some nurses in municipal healthcare do not have enough competence and skills to meet the advanced needs of healthcare for the frail elderly. Today, it is not required that nurses in the municipal healthcare have a postgraduate education specialising in care for elderly and both managers and doctors believe this is reflected in the nurses’ skills. Therefore, the managers believed that a role of ANP also could be used to educate and support other nurses and thereby also increase their competence. The participants highlighted the new healthcare agreement that started 2017 [35] with the meaning of a more advanced medical care will be performed in the patients’ homes instead of in the hospital. All participants agreed that the healthcare of the frail elderly will require high skills among nurses in municipal healthcare and thought the role of ANPs could be valuable to enable this.

A part of the new healthcare agreement is to look at the competencies issues. It is not only about ensuring that we bring in people who have the right skills but also how we use those skills. (Manager, municipal healthcare/J).

### ANP as a challenge

In this theme reflected opinions about the role of ANP as a challenge from two different perspectives, which could be seen as barriers to enabling ANPs in municipal healthcare. However, these two perspectives also include aspects that can facilitate the challenges.

### The role of ANP from the perspective of closer collaboration between municipal healthcare and primary care

The participants believed it would be a challenge to implement a role of ANP in municipal healthcare because the doctors and the municipal nurses belong to different organisations. The implementation would be easier if municipal healthcare were part of the same organisation as the primary care because the doctors would be more available for support and to hand over assignments and responsibilities to the ANP.

As they have in Sweden today with health centres [i.e. primary care doctors] and the municipal [healthcare] nurses for itself [i.e. separate organisations] if all of our nurses’ assumption from a health centre it had been easier for the doctors to release the responsibility [to an ANP] … but when in two separate organisations, I think it can raise resistance (Manager, municipal healthcare/I).

It is also important for doctors and nurses to work in closer collaboration if the role of ANP become a reality because, according to the participants, an ANP must have a doctor as a supervisor even if they were unsure if they would have enough time to be a supervisor. However, the doctors do not think it is necessary for them to act as a supervisor; instead, they would like to see ANPs as co-workers.

You had hardly needed a supervisor if you felt that you were colleagues… …or partners that could discuss the situation and how to do. (Doctor/A).

### The role of ANPs from the perspective of motivating nurses to study and the courage to take on the responsibilities that the role entails

If possible, the nurses thought it would be of interest to them to study as an ANP; however, they also mentioned certain barriers for their motivation. The nurses argued about whether it would be worth it. If it would result in a higher salary, then they thought it would be of interest, otherwise not. The managers expressed that it was the employer’s responsibility to motivate nurses to study as an ANP.

It is important that we, as employers, have a dialogue about levels of compensation. It will be worthwhile, perhaps with paid education, and when that is achieved, there is also money to collect as an incentive. (Manager, municipal healthcare/I).

The managers also emphasised the importance of clarifying what kind of tasks an ANP should have compared with a specialist or a registered nurse. If the nurses knew that they would be given other and more advanced duties after ANP training, that could be a motivator. However, the managers had some doubts about whether the nurses would be interested in training as an ANP even when compensated with a higher salary or greater responsibility. They expressed that a person must have a personal interest and engagement and considered that not many nurses reflect these qualities. The managers and the nurses emphasised that one reason was because of it is a tedious process for experienced nurses with an older education and without a Bachelor’s degree to qualify for studies at the postgraduate level. They thought that the requirements for a Bachelor’s degree could result in a lack of motivation from the nurses to study as an ANP, but nevertheless, they also thought that younger nurses who already have a Bachelor’s degree may be interested in training as an ANP if job openings for ANPs in healthcare became a reality.

Opinions were also stated about whether the nurses would consider taking he responsibility that the role of an ANP entails. The managers believed that the nurses would consider taking it on, but that was a question of education. The managers thought that if the nurses would feel that they have more competence after they have trained to become an ANP, then they would also be confident to work as one. Although the nurses stated that they would consider taking the responsibilities that the ANP role entails, the doctors were not convinced that the nurses would do this. Therefore, the doctors questioned whether this role would be of interest to the nurses if ANP positions became available.

Many [nurses] do not want to take the responsibility that it would mean [the role of ANP] … Some of you [the nurses] had made it brilliant, but many have not. (Doctor/A).

## Discussion

In general, the participants have a positive attitude regarding the role of ANPs in municipal healthcare and could see the opportunities that this role can have for meeting the increasing healthcare needs of the frail elderly. The managers and doctors mainly focused on how the doctor’s workload could be reduced by ANPs. In contrast, the nurses tended to focus more on how this role could help the frail elderly through better continuity of care and being a professional who is able to take responsibility for both their nursing needs and medical care. They emphasised that this would allow the frail elderly to feel that they are receiving person-centred care. Although the results of this study only present an assumption of how the role of ANPs could help the frail elderly, it falls in line with other studies that also focus on the patient’s satisfaction with this role [13, 36, 37].

Studies [13, 36] have shown higher patient satisfaction when visiting an ANP as compared to visiting a doctor, as the ANP can spend more time with the patient and provide a more person-centred care as emanates from the patient’s wishes. Frail elderly have complex needs for both advanced nursing and medical care, as they are in a situation of multiple diseases and dependency [30]. This requires continuity of the staff who provide care for them [2, 31], and in our study the role of ANPs is seen as a possible way to provide this. Limited continuity increases the change that the frail elderly will be treated by several doctors, where no one takes full responsibility for their medical care, leading to possible unnecessary hospitalisation. [1, 2]. The primary care should be the first line of healthcare for the frail elderly [1, 2]. However, our study shows that this fails because there are not enough doctors in the primary care system and the managers struggle with difficulties in recruiting more doctors. These difficulties are not unique to our study and confirm the results of other studies carried out globally [38, 39]. An interesting finding is that the doctors appear positive about the role of ANPs, in contrast to earlier studies [14–16] which show that many doctors feel threatened by ANPs. An interpretation of this may be that the doctors have become exhausted over the constant lack of colleagues and want a solution. Our study shows that employing ANPs is one way to solve this shortage. This result confirms the results of other studies that found that ANPs serve as an important bridge between the nursing and medical disciplines. The ANPs have a competence that complementary the doctors when handling the extensive and complex pathological picture that is commonly present with the frail elderly, and they contribute to ensuring a high quality of nursing care [27, 39].

The shortage of doctors also affects the nurse’s workload illustrated in our study by nurses expressing how tedious it is to find a doctor to consult. An interpretation of this might be to have ANPs in the municipal healthcare in comparison to having only registered and specialist nurses would make the nurses less dependent of doctors. The role of ANP have added a profession which independently of doctors can diagnose and treat more diseases of the frail elderly in their home than the nurses can do today. Consequently, this result may be of value from a view to make the healthcare more effective. In comparison, Jones et al. [40] found that employing ANPs in municipal healthcare resulted in the frail elderly being able to be cared for in their own homes instead of in hospital, making the healthcare more effective by freeing-up hospital beds. Other studies point out that ANPs reduce hospital admissions for the care of the frail elderly [40, 41].

Although our study claims that the role of ANP can be seen as important in municipal healthcare from several perspectives, there are certain circumstances that might be of hindrance to make it a reality. The greatest obstacle seems to be difficulties to motivate nurses to study to become an ANP and doubts whether they would have the courage to take on the responsibility that this role would imply. Because of this aspect, the doctors in our study not think it would be of greater interest to train to an ANP. This result corroborates with a newly published review about experiences of ANP in general practice by Jakimowicz [11], who concluded that many nurses do not want greater responsibilities. Harris & Murman [41] confirms the challenges as reveals in our study about difficulties to motivate nurses to further education and emphasise that nurses must have a personal and a professional motivation to want to develop. However, they also point out the employer’s attitude to give time to study, paid education, higher pay and extended responsibility after the studies as important conditions [42]. These challenges may be important to pay attention to if ANPs becomes a reality in a Swedish context, i.e. to create conditions so the nurses to fact choose to study to it.

### Study limitations

The study design has allowed an emphasis to be placed on the interpretations of the participant’s opinions. The method used in this analysis, content analysis [30] provided an opportunity to structure and present the result using categories and themes. By interviewing people from various professions, it was possible to capture different views of the role of ANP which might have a positive impact for the credibility of the study, although the sample is quite small (*n* = 12). According to the small sample, generalisations must be done with caution. A weakness to the study’s credibility was that the participants had different levels of earlier knowledge about the role of ANP. This may have affected the quality and depth in the interviews and might have resulted in that some became more contentious than others. There is always a risk of subjectivity in data interpretation as it is possible to interpret a text in different ways as well as it can be influenced by the interpreter’s life experience and ability. To reduce the risk of subjectivity the findings were continuously discussed by the authors during the analysis until a high degree of intersubjective agreement was reached. By presenting quotations, it is possible for the readers to judge the confirmability as they demonstrate how findings were grounded in the participant’s opinions [32].

## Conclusion

ANPs is considered to be a valuable resource not only for the frail elderly but also for the nurses in the municipal healthcare and for the doctors in primary care as they probably would make healthcare more effective. This study contributes by raising awareness about the opportunities with the role of ANP present for Swedish healthcare as this role is relatively unknown to leaders and stakeholders in the healthcare sector. Moreover, the findings are important as they represent opinions from various professionals who may help in the ongoing process of finding new ways to meet the increasing healthcare needs of the frail elderly.

## Additional files

Additional file 1: Interview guide (DOCX 14 kb)

Additional file 2: Consolidated criteria for reporting qualitative studies (COREQ): 32-item checklist. Evidence to support the quality of the reporting of the study (DOCX 15 kb)
