# Supplementary material for: Advanced nurse practitioners in municipal healthcare as a way to meet the growing healthcare needs of the frail elderly: a qualitative interview study with managers, doctors and specialist nurses
Source: BMC Nurs. 2017 Nov 16;16:63. doi: 10.1186/s12912-017-0258-7 (PMC5689167; doi:10.1186/s12912-017-0258-7)
Supplement: Supplementary file 1 — Interview guide (DOCX 14 kb) [file 12912_2017_258_MOESM1_ESM.docx]

Additional file 1: Interview Guide

Personal information

- Sex
- Age
- Profession

Questions

Did you hear about Advanced Nurse Practitioners before?

Do you know anything about what they do and what their role in healthcare is?

Studies in other countries have shown that Advanced Nurse Practitioners improve the availability, continuity of care and patient safety. What is your opinion on this matter?

Do you think that introducing Advanced Nurse Practitioners into the municipal healthcare in Sweden could possibly to solve inadequate continuity of care and doctor availability for the frail elderly?

Studies have shown that the limited authority that the nurses have when it comes to prescribing medication is a hindrance for the development of Advanced Nurse Practitioner role in healthcare. In many countries, the authority of Advanced Nurse Practitioners has been extended to include prescribing medication. What is your opinion about this issue? Do you think that extending the nurses’ authority to include the prescription of medication would also be possible in Sweden? What could the potential risks or benefits be? (Nurses received an additional question: How do you currently use your authority for medication purposes?)

What do you think about the fact that Advanced Nurse Practitioners also have the authority to order blood sampling and other tests, as well as to provide referrals for other assessment? Do you think this could also be possible in Sweden? What benefits or risks associated with this do you foresee?

Studies have shown that doctors are reluctant to have this new profession implemented in healthcare. Why do think it is like this?

Studies have shown that patients experience care that is more person-centred from Advanced Nurse Practitioners in comparison to the care provided by doctors. Why do you think it is like this?

In your opinion, would it be of interest to nurses to study for the role of an Advanced Nurse Practitioner, if this was a possibility?

What is your opinion about the competence of the nurses in municipal healthcare in relation to the increasing number of frail elderly?

What is your opinion regarding how the increasing healthcare needs of the frail elderly will be resolved in the future?

Is there anything else that you would like to add that could be of interest in this discussion?

Probing questions: How do you think? Can you please give an example?
