# Supplementary material for: Advanced nurse practitioners in municipal healthcare as a way to meet the growing healthcare needs of the frail elderly: a qualitative interview study with managers, doctors and specialist nurses
Source: BMC Nurs. 2017 Nov 16;16:63. doi: 10.1186/s12912-017-0258-7 (PMC5689167; doi:10.1186/s12912-017-0258-7)
Supplement: Supplementary file 2 — Consolidated criteria for reporting qualitative studies (COREQ): 32-item checklist. Evidence to support the quality of the reporting of the study (DOCX 15 kb) [file 12912_2017_258_MOESM2_ESM.docx]

Additional file 2: Consolidated criteria for reporting qualitative studies (COREQ): 32-item checklist

| No. Item | Guide question/Description | Response |
| --- | --- | --- |
| **Domain 1: Research team and reflexivity** |  |  |
| Personal Characteristics |  |  |
| 1. Interviewer/facilitator | Which author/s conducted the interview or focus group? | BL conducted the interviews. |
| 2. Credentials | What were the researcher’s credentials? | BL: RN, MSc.  KSF: BSc, MPH, PhD. |
| 3. Occupation | What was their occupation at the time of the study? | BL: Registered nurse. KSF: Post Doctor |
| 4. Gender | Was the researcher male or female? | Female. |
| 5. Experience and training | What experience or training did the researcher have? | BL: Nursing  KSF: Public health and health science |
| Relationship with participants |  |  |
| 6. Relationship established | Was a relationship established prior to study commencement? | Information is given in the method section. |
| 7. Participant knowledge of the interviewer | What did the participants know about the researcher? | They knew the reasons for doing the research and the affiliations of the researchers. |
| 8. Interviewer characteristics | What characteristics were reported about the interviewer/facilitator? | Reasons and interests in the research topic. |
| **Domain 2: Study design** |  |  |
| Theoretical framework |  |  |
| 9. Methodological orientation and Theory | What methodological orientation was stated to underpin the study? | Content analysis. |
| Participant selection |  |  |
| 10. Sampling | How were participants selected? | Information is given in the method section. |
| 11. Method of approach | How were participants approached? | Information is given in the method section. |
| 12. Sample size | How many participants were in the study? | It was 12 participants, ten women and two men. |
| 13. Non-participation | How many people refused to participate or dropped out? Reasons? | No one refused to participate or dropped out. |
| Setting |  |  |
| 14. Setting of data collection | Where was the data collected? | The interviews took place at the participant’s workplaces. |
| 15. Presence of non-participants | Was anyone else present besides the participants and researchers? | No. |
| 16. Description of sample | What are the important characteristics of the sample? | Majority of the participants were female. The participants consisted of four doctors, four specialist nurses and four managers. All the doctors and two of the managers worked in primary care. All the specialist nurses and two of the managers worked in municipal healthcare. |
| Data collection |  |  |
| 17. Interview guide | Were questions, prompts, guide provided by the authors? Was it pilot tested? | The interview guide is enclosed with the manuscript. |
| 18. Repeat interviews | Were repeat interviews carried out? If yes, how many? | No. |
| 19. Audio/visual recording | Did the researcher use audio or visual recording to collect the data? | The interviews were audiotaped. |
| 20. Field notes | Were field notes made during and/or after the interview or focus group? | Short field notes were made after the interviews. |
| 21. Duration | What was the duration of the interviews or focus group? | The duration of the interviews were 30-50 minutes. |
| 22. Data saturation | Was data saturation discussed? | Yes. |
| 23. Transcripts returned | Were transcriptions returned to participants for comments and/or correction? | No. |
| **Domain 3: analysis and findings** |  |  |
| Data analysis |  |  |
| 24. Number of data coders | How many data coders coded the data? | BL coded the data and did the analysis, continuously discussed with KSF. |
| 25. Description of the coding tree | Did authors provide a description of the coding tree? | Yes. |
| 26. Derivation of themes | Were themes identified in advance or derived from the data? | The themes were derived from the data. |
| 27. Software | What software, if applicable, was used to manage the data? | Not applicable. |
| 28.Participant checking | Did participants provide feedback on the findings? | No. |
| Reporting |  |  |
| 29. Quotations presented | Were participant quotations presented to illustrate the themes/findings? Was each quotation identified? | Quotations were presented and identified by profession. |
| 30. Data and findings consistent | Was there consistency between the data presented and the findings? | Yes. |
| 31. Clarity of major themes | Were major themes clearly presented in the findings? | Yes. |
| 32. Clarity of minor themes | Is there a description of diverse cases or discussion of minor themes? | Yes |

Developed from : Tong A, Sainsbury P, Craig J. Consolidated criteria for reporting qualitative research (COREQ): a 32-item checklist for interview and focus groups. *Int J Qual Health Care* 2007 19 (6) 349-357.
